# Compensatory increase in ipsilesional supplementary motor area and premotor connectivity is associated with greater gait impairments: a personalized fMRI analysis in chronic stroke

**DOI:** 10.3389/fnhum.2024.1340374

**Published:** 2024-02-29

**Authors:** Xiaolong Peng, Shraddha Srivastava, Falon Sutton, Yongkuan Zhang, Bashar W. Badran, Steven A. Kautz

**Affiliations:** ^1^Department of Psychiatry and Behavioral Sciences, Neuro-X Lab, Medical University of South Carolina, Charleston, SC, United States; ^2^Ralph H. Johnson VA Medical Center, Charleston, SC, United States; ^3^Department of Health Sciences and Research, College of Health Professions, Medical University of South Carolina, Charleston, SC, United States; ^4^Division of Physical Therapy, Department of Rehabilitation Sciences, College of Health Professions, Medical University of South Carolina, Charleston, SC, United States

**Keywords:** stroke, gait, fMRI, functional connectivity, motor rehabilitation

## Abstract

**Background:**

Balance and mobility impairments are prevalent post-stroke and a large number of survivors require walking assistance at 6 months post-stroke which diminishes their overall quality of life. Personalized interventions for gait and balance rehabilitation are crucial. Recent evidence indicates that stroke lesions in primary motor pathways, such as corticoreticular pathways (CRP) and corticospinal tract (CST), may lead to reliance on alternate motor pathways as compensation, but the current evidence lacks comprehensive knowledge about the underlying neural mechanisms.

**Methods:**

In this study, we investigate the functional connectivity (FC) changes within the motor network derived from an individualized cortical parcellation approach in 33 participants with chronic stroke compared to 17 healthy controls. The correlations between altered motor FC and gait deficits (i.e., walking speed and walking balance) were then estimated in the stroke population to understand the compensation mechanism of the motor network in motor function rehabilitation post-stroke.

**Results:**

Our results demonstrated significant FC increases between ipsilesional medial supplementary motor area (SMA) and premotor in stroke compared to healthy controls. Furthermore, we also revealed a negative correlation between ipsilesional SMA-premotor FC and self-selected walking speed, as well as the Functional Gait Assessment (FGA) scores.

**Conclusion:**

The increased FC between the ipsilesional SMA and premotor regions could be a compensatory mechanism within the motor network following a stroke when the individual can presumably no longer rely on the more precise CST modulation of movements to produce a healthy walking pattern. These findings enhance our understanding of individualized motor network FC changes and their connection to gait and walking balance impairments post-stroke, improving stroke rehabilitation interventions.

## Introduction

Balance and mobility impairments are common issues in post-stroke populations. About thirty percent of survivors are unable to walk without some assistance at the 6-month mark post-stroke (Thom et al., 2006; Asaka et al., 2008; Lloyd-Jones et al., 2009). Slower walking speed normally leads to limited community ambulation (Perry et al., 1995; Lord et al., 2004; Fulk et al., 2010), which further results in a diminished quality of life (Kaffenberger et al., 2022). Impaired balance can give rise to falls (Tilson et al., 2012; Bower et al., 2019) or fear of falls (Goh et al., 2016), and limited independence in walking (Au-Yeung et al., 2003; Mackintosh et al., 2005). Therefore, it is crucial to design effective gait and balance rehabilitation interventions tailored to the unique levels of impairment in each individual. However, due to a limited understanding of the underlying neural mechanisms of impaired balance and mobility after stroke, there remains a knowledge gap concerning how to individualize rehabilitation intervention to address specific balance and mobility deficits following stroke.

Stroke lesions of primary motor pathways may result in reliance on alternate motor pathways, which are typically associated with greater motor impairments. Corticoreticular pathways (CRP) and corticospinal tract (CST) are typically involved in movement control and muscle coordination (Smith et al., 2019; Maslovat et al., 2020). CST is involved in the control of muscle activity during walking in individuals without a neurological injury (Schubert et al., 1997; Capaday et al., 1999; Petersen et al., 2012). Animal studies have shown that pyramidal tract neurons that are the origin of the CST, modulate muscle activity during walking, and activity of pyramidal tract neurons in cats increases substantially while stepping over obstacles compared to steady state walking (Drew, 1993). Whereas the CRP has bilateral projections to the spinal cord. Therefore, CRP is likely responsible for a general motor pattern (Matsuyama et al., 2004) augmented by a more precise CST modulation of movements (Drew et al., 2004) such as negotiating obstacles or uneven terrain, seen during community walking. Previous studies on upper extremity motor function show that damage to the motor pathway by a stroke will enhance the connectivity of CRP as compensation for the reduced white matter integrity of CST, which usually accompanies a compromised motor coordination (Bradnam et al., 2013; Schulz et al., 2017; Karbasforoushan et al., 2019). Aligning with these findings, our recent study revealed that individuals who relied more on the CRP fibers as a compensatory mechanism following damage to CST on the lesioned hemisphere had more pronounced balance and mobility impairments (Srivastava et al., 2022). Considering the majority of CST fibers have their origins in the primary motor cortex (M1) whereas the CRP fibers stem from the premotor and supplementary motor area (SMA) (Jang and Seo, 2014; Jang and Lee, 2019), disrupted CST by a stroke lesion could lead to a compensatory greater reliance on alternate motor pathways responsible for a general bilateral motor pattern, with a potential tradeoff of the compensation being greater gait and balance deficits.

Recent developments in brain imaging techniques allow for investigating functional connections within specific functional networks using resting-state functional magnetic resonance imaging (rs-fMRI). Capitalizing on this approach, multiple prior studies have demonstrated that functional connectivity (FC) in SMA and premotor network plays important roles in regulating normal gait (Fukuyama et al., 1997; Hamacher et al., 2015; Lu et al., 2015; Yuan et al., 2015; Poole et al., 2019) and balance (Wittenberg et al., 2017). These functional networks are altered after a stroke. Specifically, increased FC of ipsilesional SMA (Sharma et al., 2009) and contralesional premotor cortex (Johansen-Berg et al., 2002; McPherson et al., 2018) were observed in stroke populations, and are associated with greater upper extremity motor impairment. Collectively, these findings suggest that increased FC between SMA, premotor, and other motor regions could potentially serve as a compensatory mechanism following neurological injury, however, there is no information on the relationship of gait and balance impairment with altered functional cortical connectivity following a stroke.

In the present study, our goal is to systematically investigate altered functional connections within the motor network and explore their relationship to the gait and walking balance deficits in individuals with chronic stroke. Based on previous literature we hypothesize that in comparison to healthy individuals stroke survivors will demonstrate greater connectivity in the ipsilesional SMA and premotor regions which will be negatively associated with walking balance and speed. We extracted 16 motor network-related parcels from a previously reported individualized cortical parcellation approach based on each single participant's resting-state fMRI data (Wang et al., 2015; Zhao et al., 2023). These individual motor parcels were then used as regions of interest (ROIs) to estimate the FC between brain regions within the motor network and compared between stroke participants and healthy controls. Last, we quantified the relationship between FC changes in the motor network and gait deficits (i.e., walking speed and walking balance) in the stroke population. The findings from this study will deepen our knowledge of individualized motor network FC changes and how they relate to gait and walking balance impairments following a stroke, which may pave the way for improving treatment in stroke rehabilitation.

## Methods

### Participants

The research database registry of the Center of Biomedical Research Excellence in Stroke Recovery (IRB approved for data sharing) was queried for all participants with chronic stroke (>6 months) for whom we had resting state fMRI, overground walking speeds, and functional gait assessment (FGA) scores from various studies approved by the Institutional Review Board of the Medical University of South Carolina. This study included 33 participants with chronic stroke (mean age: 63.82 ± 10.15 years; 22 males) and 17 similarly-aged healthy individuals (mean age: 58.76 ± 10.66 years; 7 males). Demographics and clinical characteristics are described in Table 1.

**Table 1:** Demographic information and clinical characteristics.

	**Control (*n* = 17)**	**Stroke (*n* = 33)**	***^#^p* value**
Age (years)	58.76 ± 10.66	63.82 ± 10.15	0.11
Gender (M/F)	7/10	22/11	0.08
Lesion side (L/R)	-	19/14	-
^*^FGA	-	18.13 ± 5.79	-
GaitSS (cm/s)	137± 23	79 ± 26	< 0.0001
FMA-LE		26.33 ± 4.51	
BBS		48.28 ± 6.83	
ABC		59.09 ± 25.94	

M, male; F, female; L, left; R, right; I, Ischemic; H, Hemorrhage; FGA, Functional Gait Assessment; GaitSS, Self-selected Speed; FMA-LE, Fugl-Meyer Lower Extremity; BBS, Berg Balance Scale; ABC, Activities-specific Balance Confidence.

^*^FGA, FMA-LE, BBS, and ABC scores have only been quantified in the stroke populations.

^#^p values were estimated using a two-sample t-test for age and GaitSS, and using a two-proportion z-test for gender.

### Clinical assessments

Clinical assessment in this study includes the Functional Gait Assessment (FGA) (Wrisley et al., 2004) used to evaluate balance during walking. It is a 10-item test where each item is scored on an ordinal scale from 0 to 3, with 0 being severe impairment and 3 being normal ambulation. It has an excellent test-retest reliability (Lin et al., 2010), as well as excellent interrater and intrareader reliability in stroke populations (Thieme et al., 2009). Participants also completed three trials of walking across a GAITRite at a comfortable walking speed (CIR Systems, Inc.; Franklin, NJ) to determine overground self-selected walking speed (Gait-SS). GAITRite is a walkway system that contains pressure sensors embedded in a roll-up mat to produce an active area 24 inches wide and 168 inches long to capture gait parameters, and has good test-retest reliability in stroke population (Kuys et al., 2011). All clinical assessments were conducted by trained physical therapists.

### MRI data acquisition

Imaging data were collected using a 3T Siemens Trio or Prisma scanner (Siemens Healthcare, Erlangen, Germany) with a 12-channel head coil. Structural MRI scans in this study include a T1-weighted imaging using an MPRAGE sequence (TR = 2,300 ms, TE = 2.26 ms, FA = 8°, FOV = 256, voxel size = 1 × 1 × 1 mm^3^) and a T2-weighted FLAIR imaging (TR = 9,000 ms, TE = 95 ms, FA = 130°, FOV = 220, voxel size = 0.4 × 0.4 × 4 mm^3^). Functional MRI data were collected using a gradient-echo echo-planar imaging sequence (TR = 2,200 ms, TE = 35 ms, FA = 90°, FOV = 192, voxel size = 3 × 3 × 3 mm^3^). All participants had one run of resting-state fMRI scan (4.5 mins) during which they were instructed to lay still, keep their eyes open, and stay awake.

### MRI data preprocessing

The fMRI data were preprocessed using a previously described analysis pipeline (Peng et al., 2023b), which included the following steps: (1) slice timing correction (Statistical Parametric Mapping, SPM2; www.fil.ion.ucl.ac.uk/spm/software/spm2/), (2) rigid body correction for head motion (FMRIB Software Library, FSL v5.0.4; https://fsl.fmrib.ox.ac.uk/fsl/fslwiki), (3) normalization for global mean signal intensity across runs, (4) bandpass filtering (0.01 to 0.08 Hz), and (5) nuisance signal regression of head-motion parameters and whole-brain, ventricular, and white matter signals.

Structural data were preprocessed using the FreeSurfer v5.3.0 software package (https://surfer.nmr.mgh.harvard.edu/). For each participant, the surface mesh of the cortical mantle was reconstructed from the structural T1-weighted image and then registered to a common spherical coordinate system. The preprocessed functional data were then registered to the FreeSurfer “fsaverage6” cortical surface template, which consisted of 40,962 vertices in each hemisphere. Spatial smoothing was performed in surface space with a 6-mm full width at half maximum Gaussian kernel.

### Lesion masks

**Figure 1:**
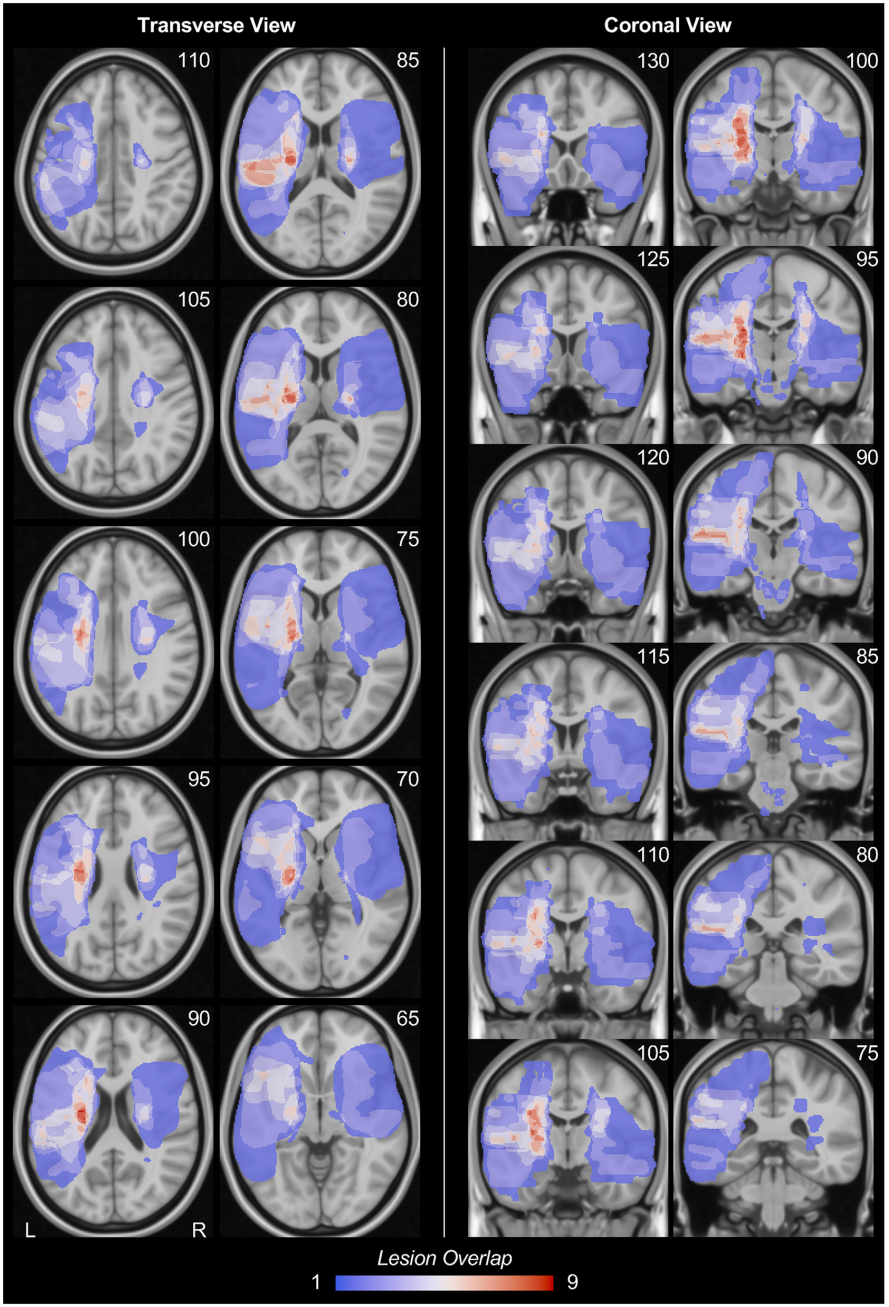
Stroke lesion overlap. The stroke lesion overlap maps were respectively displayed in the transverse view and coronal view in MNI152 space. Note that, for this lesion overlap map, all lesion masks were on their original hemisphere. The number at the upper-right corner of each figure indicates the slice coordinates. L, left hemisphere; R, right hemisphere.

Stroke lesion masks were manually drawn by a neurologist based on each participant's T2-weighted FLAIR images using MRIcron software (https://www.nitrc.org/projects/mricron). The lesion masks were then projected onto the MNI152 space through the transmission matrix derived from a co-registration between T2, T1, and the MNI152 template using SPM software. The lesion overlap map was then created by summing up the binarized lesion masks of all participants within the MNI152 space (Figure 1). Moreover, we also compared the lesion overlap map with brain segmentations derived from the Harvard-Oxford cortical and subcortical structural atlases (https://fsl.fmrib.ox.ac.uk/fsl/fslwiki/Atlases) and calculated the lesion frequency (maximum frequency) within each brain region. Participants with cortical lesions were excluded from the further analyses.

### Individualized cortical ROIs parcellation

**Figure 2:**
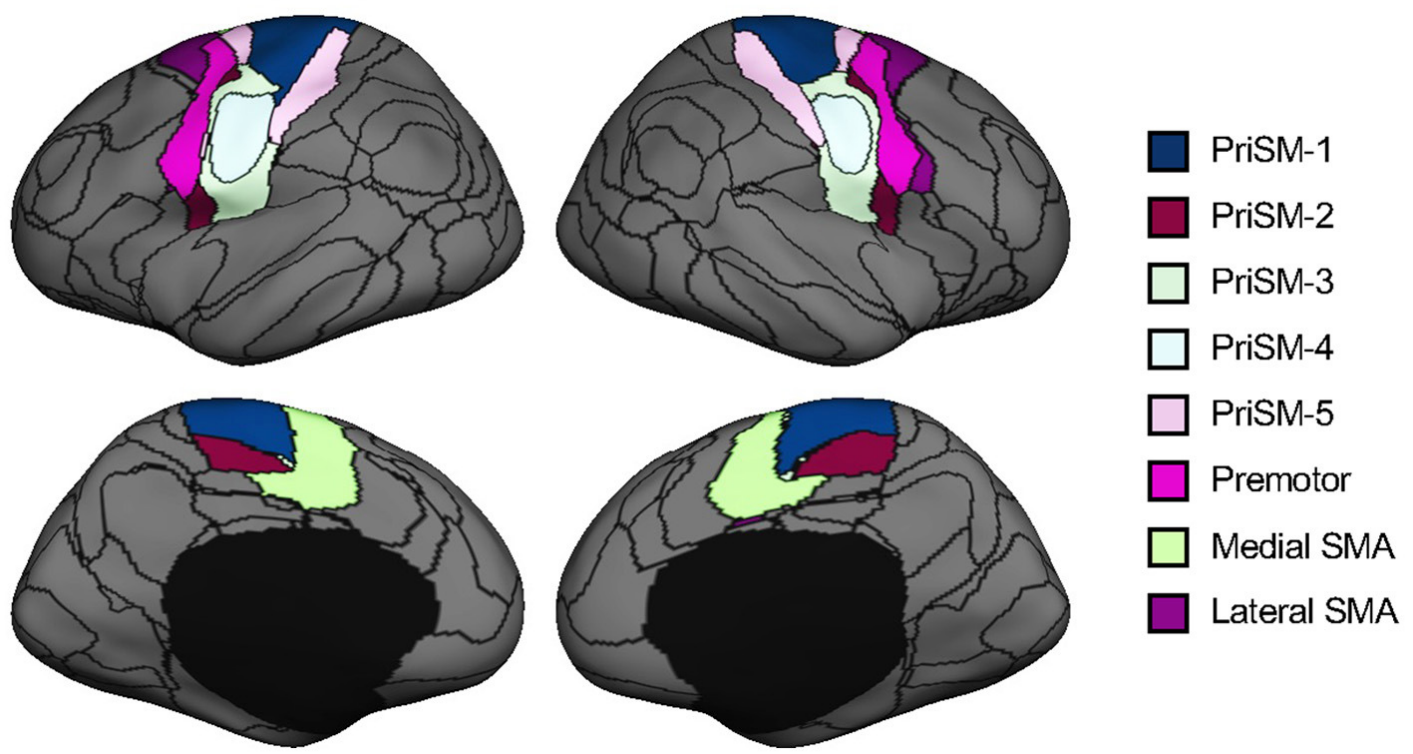
Motor-related ROIs derived from cortical parcellation. Sixteen motor-related ROIs were selected from a parcellation of the human brain into 92 ROIs. These ROIs were symmetrically located on both the left and right hemispheres. For each hemisphere, there were five primary sensorimotor (PriSM) ROIs (i.e., PriSM-1, PriSM-2, PriSM-3, PriSM-4, and PriSM-5), two supplementary motor area (SMA) ROIs (i.e., Medial SMA and Lateral SMA), and one premotor ROI (i.e., Premotor). Note that, the motor-related ROIs displayed here on the FreeSurfer fsaverage6 template are derived from a group-level cortical parcellation on 1,000 healthy individuals from the Genomic Superstruct Project for a better illustration purpose to show their locations. In this study, participant-specific ROIs, which were obtained from the individualized parcellation on each participant's brain, were applied for the FC analysis.

We parcellated each individual subject's cortex into 92 regions using an iterative approach which has been previously reported (Wang et al., 2015; Zhao et al., 2023). Briefly, this individualized cortical parcellation approach includes the following steps: (1) creating 92 group-level cortical ROIs using k-means clustering on resting-state fMRI of 1,000 healthy individuals from the Genomic Superstruct Project (Holmes et al., 2015), (2) projecting the group-level ROIs onto the individual cortex and refining the boundaries of individualized ROIs using an iterative algorithm. During the iterative algorithm, the distribution of inter-subject variability and scanning signal-to-noise ratio were applied to weight the parcellation attractors to define the individualized ROIs. In this study, 16 motor-related ROIs were selected from the 92 ROIs to investigate the motor functional network changes in chronic stroke (Figure 2). Specifically, 8 ROIs were on the left hemisphere and the other 8 ROIs were on the symmetric locations on the right hemisphere. For each hemisphere, the 8 ROIs consist of five primary sensorimotor (PriSM) ROIs (i.e., PriSM-1, PriSM-2, PriSM-3, PriSM-4, and PriSM-5), two supplementary motor area (SMA) ROIs (i.e., Medial SMA and Lateral SMA), and one premotor ROI (i.e., Premotor). FC was then estimated between these motor-related ROIs. Note that, the FC was quantified at the individual level using participant-specific ROIs, which derived from an individualized cortical parcellation method and slightly differ in shape and location across different subjects (see examples of medial SMA and premotor ROIs in Supplementary Figure S1).

### Statistical analysis

The laterality of stroke lesions plays an important role in stroke rehabilitation (Liu H. et al., 2020; Peng et al., 2023a). To control the effects of lesion laterality on motor FC changes in stroke, the FC of subjects whose stroke lesions on the right hemisphere were flipped to the left hemisphere. This procedure was to ensure all participants had their stroke lesion on the left hemisphere—the ipsilesional hemisphere. For healthy individuals, the FC analysis was carried out in their original hemisphere (i.e., the left hemisphere of healthy controls always matches the ipsilesional hemisphere for stroke, and vice versa). A Wilcoxon rank sum test was then carried out to compare the changes in FC of all motor-related ROIs between stroke and healthy populations. Lastly, the relationship between abnormal FC and gait measures was quantified using the Pearson correlation (two-tailed). To control the effect of high-leveraged data on correlation analyses, we also performed a Dfbetas analysis on the dataset used for correlation analysis. Data points with Dfbetas value larger than 3/sqrt(n) in absolute value were excluded. The findings of correlation analysis derived from both the full data and the high-leveraged points removed data were then reported. Shapiro-Wilk test of normality was conducted to determine whether speed and FGA for the stroke population are normally distributed. The results indicate that the data is normally distributed (Speed; *p* = 0.796, FGA; *p* = 0.227). All the statistical results were corrected for the multiple comparisons using the False Discovery Rate (FDR) method. All the statistical analyses were performed using MATLAB software (version: R2018b; https://www.mathworks.com/products/matlab.html).

## Results

### Stroke lesion overlap

Stroke lesion overlap maps are displayed in the MNI152 template in Figure 1. All the stroke lesions were in their original hemisphere, in which 19 participants have lesions in the left hemisphere while 14 participants have lesions in the right hemisphere. The stroke lesions are mainly located in the basal ganglia regions as well as the white matters connected to the sensorimotor cortex. The detailed lesion frequency within each brain region is shown in Table 2.

**Table 2:** Lesion frequency in different brain areas.

**Regions**	**Lesion frequency (%)**		
	**Left hemisphere**	**Right hemisphere**	**Total**
Cerebral White Matter	27.27	21.21	48.48
Cerebral Cortex	21.21	6.06	27.27
Lateral Ventrical	9.09	3.03	12.12
Thalamus	18.18	12.12	30.3
Caudate	15.15	6.06	21.21
Putamen	24.24	15.15	39.39
Hippocampus	9.09	6.06	15.15
Amygdala	9.09	6.06	15.15
Accumbens	6.06	3.03	9.09
Brain stem	-	-	12.12

### Functional connectivity of ipsilesional SMA and premotor increases in stroke

**Figure 3:**
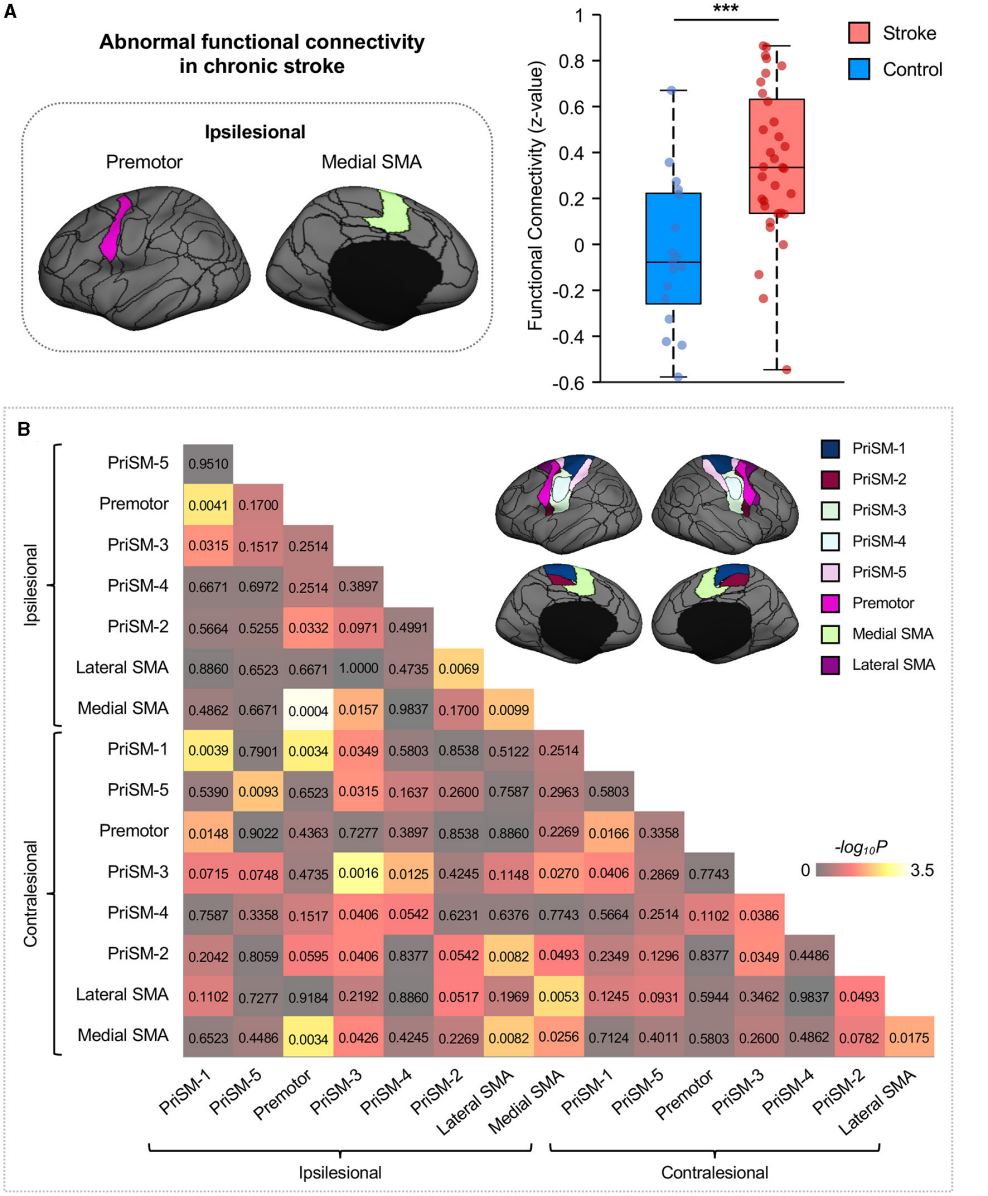
Functional connectivity between ipsilesional premotor and medial SMA increases in stroke patients. **(A)** Group comparison indicated that FC between ipsilesional premotor and medial SMA significantly increased in stroke patients compared to healthy controls (Wilcoxon rank sum test with FDR correction for multiple comparisons, ^***^p = 0.0004, *z* = 3.523). The ROIs displayed here are group-level ROIs on the FreeSurfer fsaverage6 template to illustrate the location of ROIs. **(B)** To increase the transparency of the results, FC comparisons of all eight ipsilesional motor ROIs and eight contralesional motor ROIs between stroke participants and healthy controls were calculated through the Wilcoxon rank sum test, and the *p*-values were displayed in the matrix. The color shown in the figure represents the -log_10_P to help distinguish the significance of the comparison findings.

Ipsilesional medial SMA demonstrated significantly increased FC to the ipsilesional premotor in participants with chronic stroke compared to healthy individuals (Figure 3A; Wilcoxon rank sum test with FDR correction for multiple comparisons, ^***^p = 0.0004, *z* = 3.523). To increase the transparency of the results, FC comparisons of all motor ROIs are also shown in Figure 3B.

### Increased ipsilesional SMA-premotor connection is associated with gait deficits

**Figure 4:**
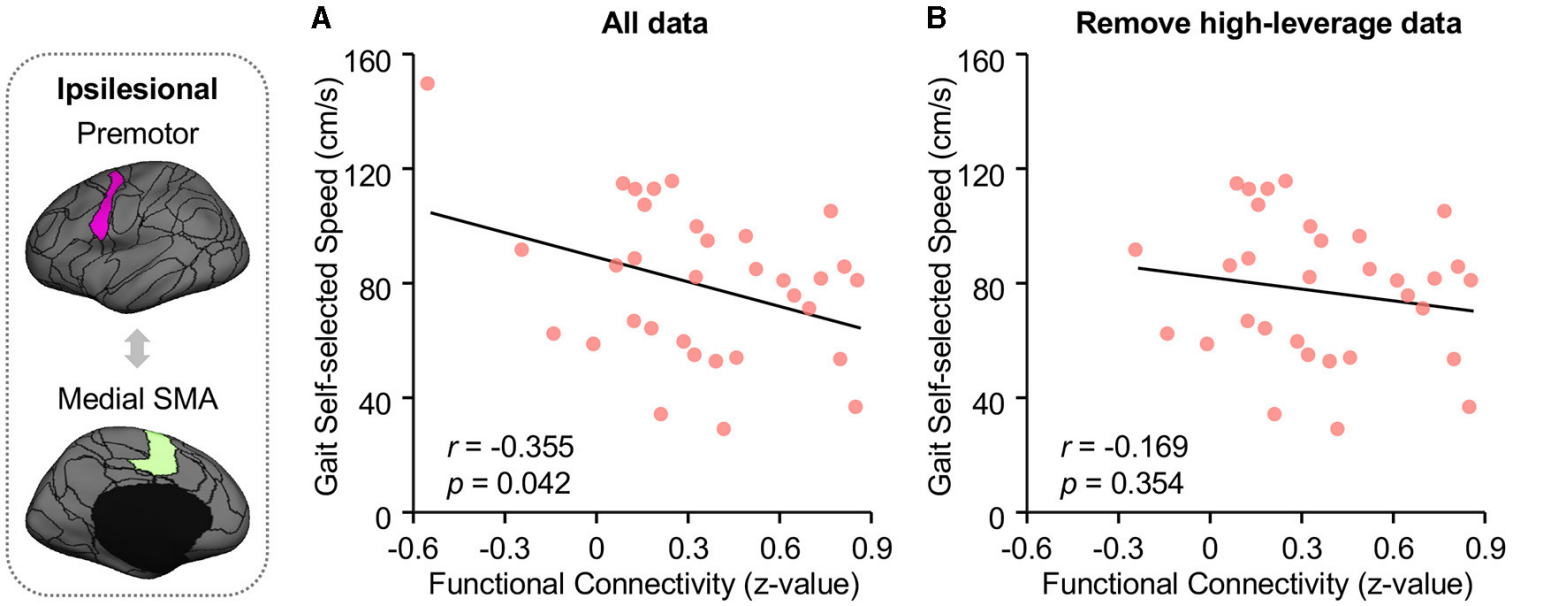
Functional connectivity of premotor and medial SMA is negatively correlated to walking speed. The walking speed of all participants was quantified at their self-selected walking speed (Gait-SS) via a GaitRite system (CIR Systems Inc., Franklin, JN). **(A)** FC between medial SMA and premotor is negatively associated with both the Gait-SS (Pearson correlation, *r* = −0.355, *p* = 0.042, 95% confidence interval [−0.623 −0.014]) across all participants with chronic stroke. **(B)** By removing the high-leveraged data points, the correlation between ipsilesional SMA-premotor FC and Gait-SS is no longer significant (*r* = −0.169, *p* = 0.354, 95% confidence interval [−0.489 −0.191]). The ROIs displayed here are group-level ROIs on the FreeSurfer fsaverage6 template to illustrate the location of ROIs.

**Figure 5:**
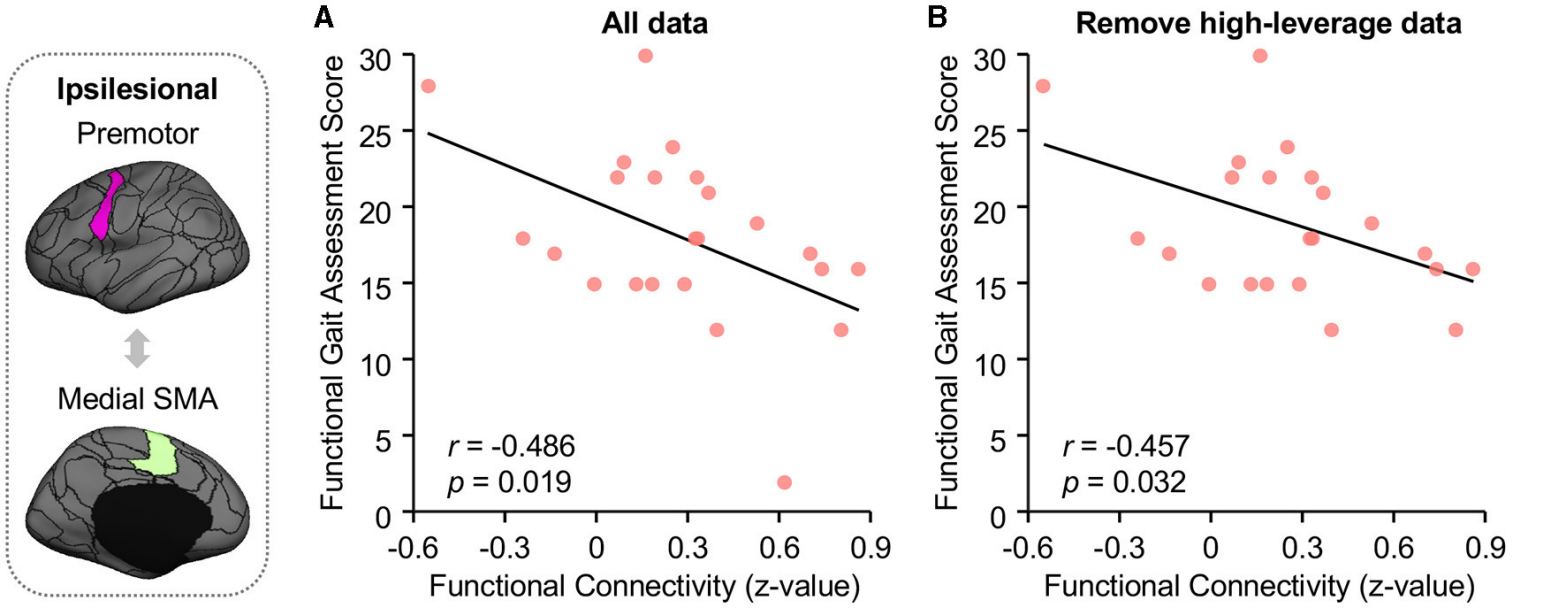
Higher functional connectivity between ipsilesional premotor and medial SMA demonstrated worse balance during walking in stroke patients. **(A)** Functional Gait Assessment (FGA) was used to assess the balance of walking in stroke patients. FC between ipsilesional premotor and medial SMA is negatively correlated to the FGA scores in stroke patients (Pearson correlation, *r* = −0.486, *p* = 0.019, 95% confidence interval [−0.748 −0.092]). **(B)** After removing the high-leveraged data points, there is still a significantly negative correlation between ipsilesional SMA-premotor FC and FGA (*r* = −0.457, *p* = 0.032, 95% confidence interval [−0.737 −0.044]). The ROIs displayed here are group-level ROIs on the FreeSurfer fsaverage6 template to illustrate the location of ROIs.

Ipsilesional SMA-premotor FC was found negatively correlated with both Gait–SS (Figure 4A; *r* = −0.355, *p* = 0.042, 95% confidence interval [−0.623 −0.014]) and FGA scores in participants with chronic stroke (Figure 5A; *r* = −0.486, *p* = 0.019, 95% confidence interval [−0.748 −0.092]). All these findings indicated that higher ipsilesional SMA–premotor FC is associated with gait deficits. Additionally, to increase the reproducibility of the findings, we also performed a control analysis by rerunning the correlation analysis with high-leveraged data points removed. The correlation between ipsilesional SMA-premotor FC and Gait-SS is no longer significant (Figure 4B; *r* = −0.169, *p* = 0.354, 95% confidence interval [−0.489 −0.191]) while there is still a significantly negative correlation between ipsilesional SMA–premotor FC and FGA scores (Figure 5B; *r* = −0.457, *p* = 0.032, 95% confidence interval [−0.737 −0.044]). These findings indicated that the high-leveraged data points may potentially affect the correlation analysis between FC and Gait-SS, which is recommended to be further verified by future clinical trials with a larger sample size and broader motor impairment data.

## Discussion

In this study, we investigated the altered FC within the motor network in chronic stroke and quantified its relationship to walking ability and walking balance. Our results demonstrated significant FC increases between ipsilesional medial SMA and premotor in stroke compared to healthy controls. Additionally, we also observed a negative correlation between ipsilesional SMA-premotor FC and self-selected walking speed, as well as the FGA scores. These findings align with our initial hypothesis suggesting that an increase in FC between the ipsilesional SMA and premotor regions could serve as a compensatory mechanism within the motor network following a stroke when the individual can presumably no longer rely on the CST to produce the healthy walking pattern. Additionally, this compensation seems stronger (higher level of ipsilesional SMA-premotor FC increase) in individuals who exhibit more severe impairments in gait and walking balance performance.

Enhanced FC between ipsilesional SMA and premotor cortex may suggest the increased contribution of alternate motor pathways as a consequence of CST damage by stroke lesion. Previous literature on white matter integrity of motor pathways has shown that compared to healthy controls, individuals with stroke who have decreased CST integrity also have increased integrity of motor pathways originating from ipsilesional SMA and premotor cortex (Rüber et al., 2012). Furthermore, individuals who have greater damage to the CST demonstrate increased structural connectivity between the primary motor network and premotor area (Schulz et al., 2017), suggesting a greater reliance on the premotor network that is dependent on the CST lesion load. The results from the current study on FC of the motor network further support the view that ipsilesional SMA and premotor cortex contribute toward motor recovery as a compensatory mechanism following a stroke lesion.

A compensatory increase in the SMA-premotor region connections likely associated with a larger CRP-modulated locomotor control leads to a gross muscle coordination pattern and worse locomotor performance. In the current study, an increased FC between SMA and premotor cortex led to slower walking speeds and worse walking balance performance perhaps reflecting a reliance on alternative motor pathways following CST damage. CRP plays an important role in controlling gross motor activities including walking and postural movements (Matsuyama et al., 2004). Increased connectivity of CRP is associated with gross muscle synergies such as mass flexion-extension patterns (Li et al., 2019), limiting the ability to independently activate muscles out of a mass flexion-extension synergy. The use of gross motor synergies leads to poor locomotor (Clark et al., 2010) and balance (Allen et al., 2019) performance in individuals with stroke. As discussed earlier, CRP tracts originate primarily from the premotor cortex and SMA (Jang and Lee, 2019), thus, we believe that increased connectivity of ipsilesional SMA-premotor regions in the current study is indicative of a greater CRP contribution in locomotor control. This likely leads to the use of mass flexion-extension patterns instead of the independent muscle activation patterns typically seen in healthy individuals (Clark et al., 2010), thus resulting in slower walking speeds and poor walking balance control represented by lower FGA scores.

The converging evidence between our results in the current study and the previous structural MRI study of gait and balance (Srivastava et al., 2022) suggests additional support for our findings. These studies will provide novel insights toward future experimental paradigms to use rs-fMRI as a biomarker for patient stratification for brain stimulation protocols. Despite the promising effects of cortical stimulation on the improvement of lower limb motor function or walking balance (Madhavan and Shah, 2012; Chang et al., 2017), due to the large variability in stroke lesions and remodeling of neural networks following stroke, there is no consensus on what cortical electrode placements (Seamon et al., 2022) or stimulation parameters (Kindred et al., 2020) would be most effective. Previous studies on upper extremity motor recovery after stroke have shown that greater ipsilesional (Sharma et al., 2009) or contralesional functional activation in regions corresponding with CRP motor pathways i.e., SMA and premotor cortex is associated with worse functional outcomes (Johansen-Berg et al., 2002; McPherson et al., 2018). Increased contralesional SMA activity associated with greater lower extremity strength was also observed by Enzinger et al. (2008). Additionally, stroke survivors with a complete CST and CRP injury and inability to walk at the time of stroke can regain their walking ability following increased contralesional CRP connectivity (Jang et al., 2013; Jang and Cho, 2022). We believe that in the event of a complete CST and CRP lesion leading to complete loss of motor function, stroke survivors would compensate by mostly relying on contralesional CRP and would likely walk with poor walking ability. Therefore, similar to our interpretation of the connectivity of motor pathways, we speculate that with ipsilesional M1 damage, ipsilesional SMA and premotor cortex gain more importance in modulating motor function. But in the event that ipsilesional M1 as well as SMA and premotor cortex are unable to function appropriately, motor control shifts more toward contralesional SMA and premotor cortex as a compensatory mechanism. Another important consideration is the altered interhemispheric interaction following stroke. This mechanism of cortical reorganization suggests that in individuals with greater damage to cortical structures, SMA and premotor cortex have a facilitatory influence on the ipsilesional M1, and this influence is inhibitory in individuals with minimal damage to promote maximal motor recovery (Di Pino et al., 2014). Additionally, although we flipped the lesion of all stroke participants to the same side in the current study to control the effects of lesion laterality on motor FC changes, it is worth noting that some other studies suggested that the laterality of stroke lesions is not always discriminant for motor rehabilitation (Chae and Zorowitz, 1998; Chen et al., 2000, 2003; Glymour et al., 2007). Future studies on individuals with mild, moderate, and severe motor impairment are needed to completely understand the interplay between lesion size/location, interhemispheric interaction, and compensatory remodeling. In addition, experimental paradigms for gait and walking balance rehabilitation that pair neuromodulation with diffusion and rs-fMRI to develop an individualized treatment should be pursued.

### Limitations

An advantage of rs-fMRI is that it can be performed in stroke survivors with severe deficits to evaluate brain networks, therefore, it has been identified as a developmental priority in stroke recovery and rehabilitation (Boyd et al., 2017). However, there are some limitations in the current study that need to be considered while interpreting the results. First, although rs-fMRI scans in the current study were collected for a duration of 4.5 min (and it would be ideal to have a longer recording), these results serve as preliminary information for future studies with longer rs-fMRI scans. Another limitation is that participants in the current study on average had mild to moderate locomotor impairment (Gait-SS = 79 ± 26 cm/s). Note that, there is one post-stroke participant who has better motor performance than others, which could potentially affect the results of the correlation analysis. It would be ideal to have a larger sample size in future clinical trials by enrolling more post-stroke individuals with either more severe or less motor impairments to better depict the relationship between motor performance and cortical functional connectivity changes. Moreover, a small percentage of CST fibers also originate from the premotor area (Liu J. et al., 2020), so there is a possibility that an increase in SMA-premotor regions could be a consequence of increased CST fibers from alternate cortical regions besides the primary motor cortex. However, we believe this would not result in worse locomotor performance as is the case with relying on alternating motor pathways such as CRP. Lastly, impaired walking speeds and poor balance can also be associated with factors other than cortical connectivity such as cognitive impairment (Ursin et al., 2019), spasticity (Soyuer and Öztürk, 2007), and altered connectivity in the subcortical regions (Qin et al., 2022) following stroke. However, a comprehensive identification and examination of the aforementioned limiting factors for walking speed and poor walking balance is beyond the scope of this study and needs to be addressed in future studies.

## Data availability statement

The original contributions presented in the study are included in the article/Supplementary material, further inquiries can be directed to the corresponding author.

## Ethics statement

The studies involving humans were approved by Institutional Review Board of the Medical University of South Carolina. The studies were conducted in accordance with the local legislation and institutional requirements. The participants provided their written informed consent to participate in this study.

## Author contributions

XP: Conceptualization, Data curation, Formal analysis, Investigation, Methodology, Supervision, Visualization, Writing – original draft, Writing – review & editing. SS: Conceptualization, Data curation, Investigation, Methodology, Writing – original draft, Writing – review & editing. FS: Investigation, Writing – review & editing. YZ: Investigation, Writing – review & editing. BB: Conceptualization, Funding acquisition, Investigation, Writing – review & editing. SK: Conceptualization, Formal analysis, Funding acquisition, Investigation, Project administration, Resources, Supervision, Writing – review & editing.
