# Supplementary material for: Compensatory increase in ipsilesional supplementary motor area and premotor connectivity is associated with greater gait impairments: a personalized fMRI analysis in chronic stroke
Source: Front Hum Neurosci. 2024 Feb 29;18:1340374. doi: 10.3389/fnhum.2024.1340374 (PMC10937543; doi:10.3389/fnhum.2024.1340374)
Supplement: Supplementary file 1 [file Image_1.pdf]

Supplementary Materials for

Compensatory increase in ipsilesional supplementary motor area and premotor connectivity is associated with greater gait impairments: a personalized fMRI analysis in chronic stroke

Xiaolong Peng, Shraddha Srivastava, Falon Sutton, Yongkuan Zhang, Bashar W. Badran, Steven A. Kautz

<u>This PDF file includes one supplemental figure referenced in the manuscript:</u>

Figure S1

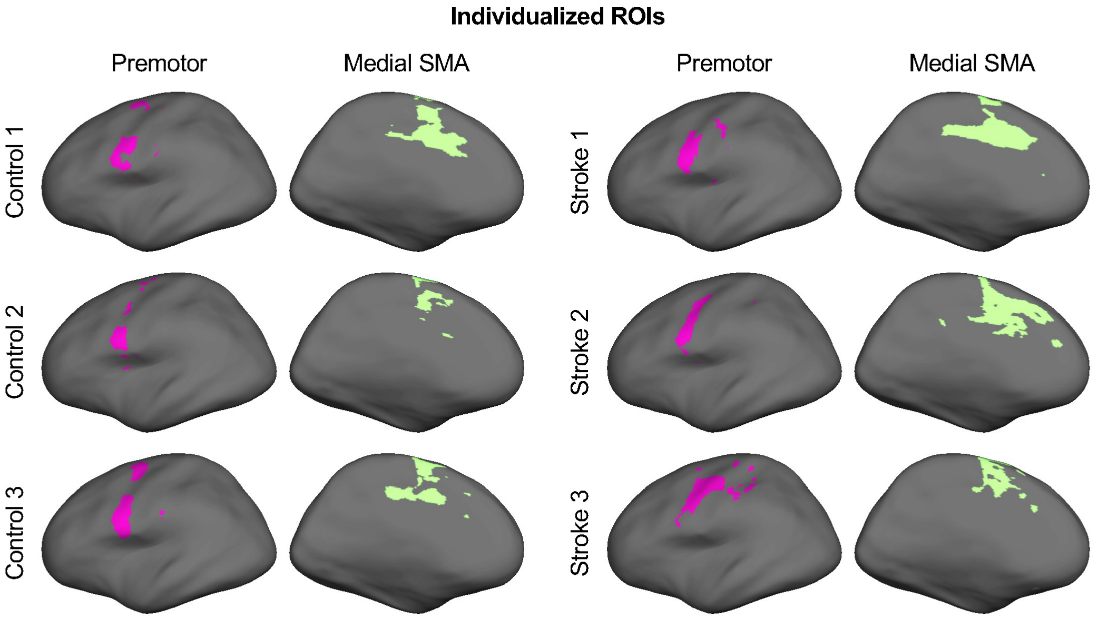

Figure S1. Individualized ROIs of premotor and medial SMA. In this study, participant-specific ROIs were applied to quantify the FC of motor-related brain regions in both stroke and healthy controls. These ROIs were generated using an individualized cortical parcellation method based on each subject's resting-state fMRI data. Here, three healthy controls and three participants with chronic stroke were randomly selected, and their individualized ROIs of premotor and medial SMA were displayed. These examples indicated that the shape and location of individualized ROIs slightly differ across different participants.
